# Mediating role of impaired wisdom in the relation between childhood trauma and psychotic-like experiences in Chinese college students: a nationwide cross-sectional study

**DOI:** 10.1186/s12888-022-04270-x

**Published:** 2022-10-21

**Authors:** Jiamei Zhang, Zhening Liu, Yicheng Long, Haojuan Tao, Xuan Ouyang, Guowei Wu, Min Chen, Miaoyu Yu, Liang Zhou, Meng Sun, Dongsheng Lv, Guangcheng Cui, Qizhong Yi, Hong Tang, Cuixia An, Jianjian Wang, Zhipeng Wu

**Affiliations:** 1 Second Xiangya Hospital, Institute of Mental Health, Central South University, Changsha, Hunan China; 2 China National Clinical Research Center on Mental Disorders, Changsha, Hunan China; 3 Department of Psychiatry, Jining Medical University, Jining, Shandong China; 4 Department of Mental Health, the Second Affiliated Hospital of Guangxi Medical University, Nanning, China; 5 Department of Social Psychiatry, the Affiliated Brain Hospital of Guangzhou Medical University, Guangzhou, Guangdong China; 6 Department of Mental Health Institute of Inner Mongolia Autonomous Region, The Affiliated Mental Center of Inner Mongolia Medical University, Inner Mongolia, Hohhot, China; 7 Department of Psychiatry, Qiqihar Medical University, Qiqihar, Heilongjiang China; 8 Psychological Medicine Center, Xinjiang Medical University, Urumqi, Xinjiang China; 9 Department of Psychiatry, Gannan Medical University, Ganzhou, Jiangxi China; 10 Department of Psychiatry, Hebei Medical University, Shijiazhuang, Hebei China; 11 Clinical Nursing Teaching and Research Section, The second Xiangya Hospital of Central South University, Changsha, Hunan China

**Keywords:** Childhood trauma, Wisdom, Mediating effect, Psychotic-like experiences

## Abstract

**Background:**

The association between childhood trauma (CT) and psychotic-like experiences (PLEs) is well-established. Many previous studies have recognized wisdom as a protective factor for mental health, but its role in the relation between CT and PLEs remains unknown. We aimed to investigate the mediating effect of wisdom in the above association among Chinese college students.

**Methods:**

We conducted a nationwide survey covering 9 colleges across China and recruited a total of 5873 students using online questionnaires between September 14 and October 18, 2021. Convenience sampling was adopted. We employed the San Diego Wisdom Scale (SD-WISE), the Childhood Trauma Questionnaire (CTQ-28), and the 15-item Positive Subscale of the Community Assessment of Psychic Experiences (CAPE-15) to measure the wisdom, CT and PLEs, respectively. Descriptive, correlation, and mediation analysis were utilized.

**Results:**

The positive correlation between CT and PLEs was well-replicated among college students (Pearson’s r = 0.30, p < 0.001). Wisdom was negatively associated with CT (Pearson’s r = − 0.46, p < 0.001) and frequency of PLEs (Pearson’s r = − 0.25, p < 0.001). Total wisdom scores partially mediated the relationship between cumulative childhood trauma, neglect, abuse and PLEs, separately. The mediated model respectively explained 21.9%, 42.54% and 18.27% of the effect of CT on PLEs. Our model further suggested that childhood trauma could be related to PLEs through decreasing the following wisdom components: decisiveness, emotional regulation and prosocial behavior.

**Conclusion:**

For the first time, our results suggested that impaired wisdom played a role in the translation from childhood adversity to subclinical psychotic symptoms, implicating wisdom as a possible target for early intervention for psychosis among young individuals. Longitudinal work is warranted to verify the clinical implications.

## Background

Childhood trauma (CT) or adversity is defined as the physical, sexual, or emotional abuse or neglect of a child, especially by parents or other caregivers. CT is a serious, worldwide public health problem which is closely related to the mental health of the youth [1]. Studies have shown that CT is associated with diverse mental illnesses including major depressive disorder [2], bipolar disorder [3] and psychosis [4–6], indicating a broad association of CT with psychopathology rather than with a single disorder. In the non-clinical population, CT has also been demonstrated to be associated with numerous mental health problems, including the well-studied psychotic-like experiences (PLEs) [7].

PLEs are generally considered to resemble positive symptoms which including delusions and hallucinations of psychotic disorders but do not reach the threshold of clinical diagnosis [8–12]. It is suggested that those who reported PLEs have a higher risk of developing clinical psychosis [13]. Previous studies [14–16] have been conducted on the relationship between CT and psychosis, all of which indicated that CT was associated with an increased risk for psychosis spectrum. Consequently, tackling the mechanism between CT and PLEs carries undeniable significance, considering the PLEs as a possible risk state for clinical psychosis and other psychiatric disorders [17–20].

Multiple factors have contributed to the relationship between CT and subsequent PLEs so far. Psychological and cognitive factors, for example, aberrant salience and self-disturbances [21] have been found to be mediators of the relationship between CT and PLEs in the general population. There are also studies showing the disruption in sleep dissociation [7], ontological insecurity [22], stress sensitivity [23] and cognitive vulnerability [24] involved in this relationship. Moreover, cognitive biases play important roles in the relationship between childhood traumatic events and the development of PLEs in non-clinical young adults [25]. These previous works provided important insights into the mechanism between CT and PLEs, but many of them adopted community-based approach and often included a small sample, which limited the generalizability of their results. Further investigation into the mediating factors, especially those suitable for interventions, in the association between CT and PLEs is warranted.

Wisdom, a neurobiological personality trait, which includes emotional regulation, self-reflection, prosocial behavior, decisiveness, social advice, and tolerance and spirit for different values [26–28], is an ancient psychological construct with a long history [29]. Empirical research of wisdom has been widely conducted since the 1970s. Moreover, it has been shown that increasing in wisdom can ease a number of mental health problems by means of improved personal well-being and happiness [30], but negatively associated with adverse physical and mental outcomes [31, 32], suggesting a protective role of wisdom to health. Furthermore, several studies showed that childhood maltreatment was significantly associated with some aspects of wisdom, for instance, prosocial behaviors [33] and emotional regulation [34], and predicted less self-reflection [35]. The commonly applied tools to measure wisdom includes the Self-Assessed Wisdom Scale (SAWS) [36], the Three-Dimensional Wisdom Scale (3D-WS) [37, 38] and the San Diego Wisdom Scale (SD-WISE) [39]. Of note, the SD-WISE, including six dimensions of wisdom, is the first wisdom measuring tool developed by psychiatrists and based on possible wisdom-related neurobiological mechanisms after reviewing many cases with brain lesions [39]. Some aspects of wisdom were previously found to be related to psychosis risks. For instance, self-reflection was similarly impaired in participants with psychosis proneness compared to patients with clinical psychosis [40]. The cognitive model of psychosis suggests that multiple form of cognitive biases, such as jump to conclusion and aberrant salience, played important roles in the maintenance of psychotic symptoms [41]. Difficulties with emotion regulation was found to be associated with the frequency and distress caused by psychotic experiences [42]. However, the association of PLEs and other components of wisdom, such as decisiveness, were still understudied.

Previous studies have found that childhood trauma has been shown to predict the onset and persistence of PLEs [43, 44] and is associated with some dimensions of wisdom as previously mentioned. According to previous research, wisdom can exert profound positive effects on life satisfaction [45], which is closely related to individual’s well-being [46] and mental health [47]. Proposed hypothesis highlighted that higher levels of specific components of wisdom may help alleviate the negative effects of physical decline [48], further improve personal happiness. Furthermore, some aspects of wisdom, such as emotional regulation and self-cognition have been studied as mediators in the relation between CT and PLEs [49, 50] by damaging the concurrent psychopathology [50].

Further, given that wisdom is amenable to interventions [27] and plays an important role in an individual’s mental health [51], it is still unclear whether wisdom can reduce the emergence of distressing PLEs, especially among those impacted by childhood trauma. Meanwhile, studies that specifically investigate the role of wisdom in the mental health of college students are still lacking. College students are in their early stage of adulthood, which is the main risk age stage of psychotic disorders [52]. For this reason, examining the role of wisdom in the relation between CT and PLEs might implicate possible targets for the early prevention of clinical psychosis among those suffered from childhood adversity.

In the current study, we addressed the above concerns and recruited a large sample of college students from 9 universities across China to investigate the mediating role of wisdom in the association between CT and PLEs in early adulthood. Specifically, we explored several hypotheses and expected to see: (1) the positive correlation between CT and PLEs is well-replicated in Chinese college students. (2) total wisdom and its different components have protective effects for PLEs and are negatively correlated with CT and PLEs. (3) wisdom moderated the relation between CT and the frequency of PLEs. We further explored this moderation of six putative components of wisdom on the relation between childhood trauma (neglect and abuse) and PLEs to better implicate future intervention for psychosis in young adulthood.

## Methods

### Participants

A set of online questionnaires was built using the Questionnaire Star Platform (www.wjx.com). The online-based questionnaires were distributed to students through social media platforms by the school officers of different institutions, which included university students from Changsha in Hunan Province, Guangzhou in Guangdong Province, Nanning in Guangxi Province, Ganzhou in Jiangxi Province, Shijiazhuang in Hebei Province, Urumqi in Xinjiang Uygur Autonomous Region, Hohhot in Inner Mongolia Autonomous Region, Jining in Shandong Province and Qiqihar in Heilongjiang Province. The nine universities have a total undergraduate population of about 130,000. Among them, a total of 5993 undergraduates were recruited for this study. Participants who had been diagnosed with any psychiatric diseases (120 cases) were excluded, leaving a total N = 5873 (98.0% retained) completed surveys that were used for descriptive analyses.

### Measurements

#### Childhood trauma questionnaire (CTQ)

The Chinese version of the Childhood Trauma Questionnaire (CTQ) was administered to assess traumatic events during childhood [53, 54], a 28-item self-report inventory assessing five types of traumas experienced by a child or teenager: emotional abuse, physical abuse, sexual abuse, emotional neglect and physical neglect. Items were scored on a scale of 1–5 (1 = never to 5 = always). Total and subscale scores were calculated, with higher scores indicating higher severity of childhood trauma. In the present study, the CTQ total score displayed good internal consistency (Cronbach’s α = 0.869).

#### 15-item Positive subscale of the community assessment of psychic experiences (CAPE-P15)

CAPE-P15 is designed to measure the frequency and distress associated with commonly-reported psychotic experiences [55]. The scale has 15 items covering the following three domains: persecutory ideation (PI), bizarre experiences (BEs), and perceptual abnormalities (PAs) [56–58]. The CAPE-15 has two subscales: the frequency of PLEs and their associated distress. The frequency subscale was adopted in our work. Each item is marked on a scale of 1–4, from 1 = never to 4 = almost always. The Chinese version of CAPE-P15 has shown good reliability [59]. The total frequency score of each subject was computed and showed good internal consistency (Cronbach’s α = 0.908).

#### San Diego wisdom scale (SD-WISE)

SD-WISE is consisted of 24 items, assessing human wisdom from the following six domains: social advising (insight), emotional regulation, prosocial behaviors, self-reflection, acceptance of divergent perspectives (tolerance), and decisiveness [60]. A greater score on the SD-WISE corresponds to greater levels of wisdom (total score range = 1–5). We validated the six-factor structure of SD-WISE using a sample of 900 college students in a prior study. The relevant article is currently being under review for publication. The Chinese version of the SD-WISE in this sample has good reliability (Cronbach’s α = 0.781).

### Data analysis

First, descriptive analyzes (N = 5873) were performed to describe the sample characteristics. Second, Pearson’s correlation was conducted between three variables to explore the bivariate interrelation between CT, PLEs and wisdom. Third, we examined the role of wisdom in the relationship between CT and PLEs. Participants with missing values in demographic data were eliminated from the analysis, leaving a total of 5835 individuals for correlation and mediation analysis. Considering abuse and neglect may contribute to PLEs in different manner, separate mediation models between CT and PLEs were also established for two traumatic domains [21]. According to Baron and Kenny [60, 61], a mediation model includes the following four steps, the first step is to check the independent variable (CT) whether has an influence on the final outcome (frequency score of PLEs). The second step is the effect between the independent variable (CT) and the proposed mediator (wisdom). The third step is to assess the relationship between the proposed mediator (wisdom) and the dependent (frequency score of PLEs) after control the independent variable (CT). The last step is to check if the effect between the independent (CT) and the dependent variable (frequency score of PLEs) is reduced (= partial mediation) after controlling the proposed mediator (wisdom). According to the procedures outlined by Baron and Kenny, the mediating effect is established only when the second and the third steps are statistically significant, the 95% CI did not contain zero, and the last steps are less significant.

We hypothesized that wisdom is negatively associated with CT and PLEs, and wisdom further affect the relationship between CT and PLEs. Three mediation models were performed to examine the role of wisdom in the relation between total subtypes (cumulative childhood trauma, childhood abuse and childhood neglect) of CT and the frequency of PLEs. Moreover, we built separate mediation analysis to assess the effects of six wisdom components on the relation between the cumulative trauma and PLEs frequency, for different wisdom factors may have different effects on the association between CT and PLEs. In addition, compared to men, women are at significantly higher risk of certain forms of trauma, such as sexual abuse [62, 63]. Further, sex has been investigated differences in the expression of PLEs between males and females. Females also seem to be more likely to show positive symptoms such as hallucinations and age was reported to associated with the wisdom level [64, 65]. To avoid the potential confounding effects, sex and age were considered as covariates in the mediation analysis. The significance effect of the third step was derived based on a bias-corrected bootstrap confidence interval (CI) based on 5000 bootstraps, which were reported if 95% CI did not cover zero.

We analyzed the data using SPSS Version 25.0. Model 4 [66] from the PROCESS macro for Windows [67] was utilized to build our mediation models.

## Results

### Descriptive analysis

One hundred and twenty participants were excluded for self-reported and any history of mental illnesses, a total of 5873 adolescents entered descriptive analysis. The mean age of our participants was 19.36 (S.D. = 1.486) and 57.33% were females. Other details of our sample’s characteristics were presented in Table 1.

**Table 1:** Sample characteristics (N = 5873)

	M	SD	Range	Number of items
Sex (F/M)	3367/2506	-	-	-
Age	19.36	1.486	16–30	-
CTQ-28, Sum	37.52	12.696	25–97	28
Abuse	18.80	7.963	15–75	15
Neglect	18.72	6.842	10–43	10
CAPE-P15	19.25	4.210	15–47	15
SD-WISE, Sum	84.14	10.591	49–120	24
Decisiveness	12.65	2.494	4–20	4
Emotional regulation	13.47	2.367	4–20	4
Self-reflection	14.79	2.338	7–20	4
Prosocial behavior	14.83	2.196	4–20	4
Insight	13.47	2.381	4–20	4
Tolerance	14.93	2.885	4–20	4

Note: CTQ-28-Sum (Cumulative Trauma) – total score of the CTQ; Neglect – a summary of physical and emotional neglect subscales of the CTQ; Abuse – a summary of sexual, physical and emotional abuse subscales of the CTQ; SD-WISE- total score of the SD-WISE; CAPE-P15- total score of the frequency subscales of the PLEs.

### Pearson’s correlation

Correlation results showed that cumulative childhood trauma (r = 0.30, P < 0.001), childhood neglect (r = 0.21, P < 0.001) and childhood abuse (r = 0.29, P < 0.001) were all significantly positively correlated with the frequency of PLEs, while the total score of CTQ was also negatively related to SD-WISE (r = − 0.46, p < 0.001) and its subscales. Meanwhile, negative correlations were observed between PLEs and SD-WISE total score (r = − 0.25, p < 0.001), as well as the components of wisdom. Details of the coefficients were presented in Table 2.

**Table 2:** Correlational matrix (N = 5835)

	CTQ-28-Sum	Abuse	Neglect	CAPE-P15	DC	ER	SR	PB	IS	TC		SD-WISE
CTQ-28-Sum	1											
Abuse	0.88	1										
Neglect	0.83	0.47	1									
CAPE-P15	0.30	0.29	0.21	1								
DC	-0.14	-0.12	-0.13	-0.26	1							
ER	-0.29	-0.19	-0.31	-0.33	0.49	1						
SR	-0.42	-0.29	-0.44	-0.09	0.27	0.42	1					
PB	-0.43	-0.29	-0.46	-0.23	0.32	0.54	0.58	1				
IS	-0.31	-0.16	-0.39	-0.11	0.24	0.42	0.50	0.40	1			
TC	-0.40	-0.22	-0.49	-0.07	0.06	0.46	0.62	0.58	0.57	1		
SD-WISE	-0.46	-0.29	-0.51	-0.25	0.54	0.76	0.78	0.78	0.72	0.77		1

All of p < 0.001

Decisiveness DC, Emotional regulation ER, Self-reflection SR, Prosocial behavior PB, Insight IS, Tolerance TC.

### Mediation

#### Cumulative childhood trauma to PLEs

**Fig. 1:**
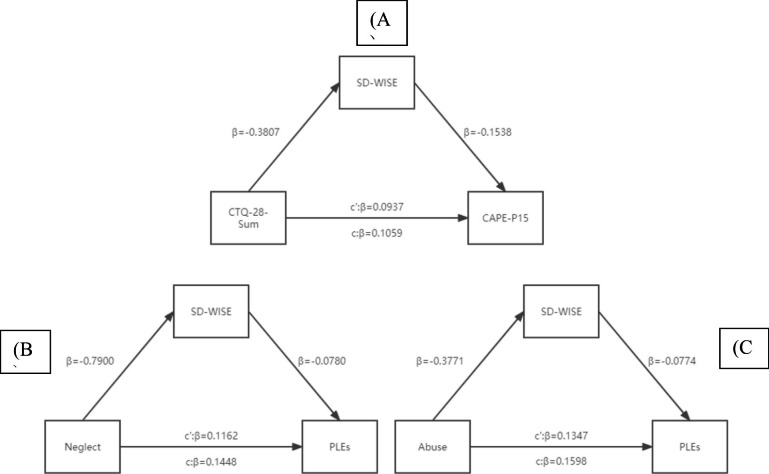
Mediation models (N = 5835). (A) The model of the relationship between cumulative childhood trauma (CTQ-28-Sum) and psychotic-like experiences (total score of the frequency). Indirect-partial mediation was found. The mediated model explained 21.90% to the effect of CTQ-28-Sum on PLEs. (B) The model of the relationship between childhood neglect (Neglect) and psychotic-like experiences (total score of the frequency). Indirect-partial mediation was found. The mediated model explained 42.54% to the effect of Neglect on PLEs. (C) The model of the relationship between childhood abuse (Abuse) and psychotic-like experiences (total score of the frequency). Indirect-partial mediation was found. The mediated model explained 18.27% to the effect of Abuse on PLEs. Gender and age were included as covariates in the analysis.

As displayed in Fig. 1, the effect of cumulative trauma on PLEs was 0.1059 (SE = 0.0042, 95% CI [0.0976, 0.1142]), the effect of wisdom on PLEs after control CT was 0.0122 (SE = 0.0027, 95% CI [0.0068, 0.0173]) and the effect of CT on PLEs after control wisdom was 0.0827 (SE = 0.0047, 95% CI [0.0735, 0.0918]), which became less but did not cross zero, indicated that wisdom partially mediated the relationship between cumulative childhood trauma and PLEs. In addition, the ratio of investigating effect over the effect of cumulative trauma on PLEs was 21.9%. We noticed that decisiveness (SE = 0.0007, 95% CI (0.0039, 0.0068)), emotional regulation (SE = 0.0019, 95% CI (0.0234, 0.0311)) and prosocial behavior (SE = 0.0024, 95% CI (0.0085, 0.0179)) were all significant mediators.

#### Childhood neglect to PLEs

The effect of childhood neglect on PLEs was 0.1448 (SE = 0.0080, 95%CI [0.1291,0.1605]) and the effect of Childhood neglect on PLEs after control wisdom was 0.0832 (SE = 0.0091, 95%CI [0.0653, 0.1010]), which did not cover zero, wisdom partly mediated the relationship between childhood neglect and PLEs. The mediated model explained 42.54% of the model effect. Similarly, we noticed that decisiveness (SE = 0.0014, 95% CI (0.0066, 0.0120)), emotional regulation (SE = 0.0038, 95% CI (0.0488, 0.0634)) and prosocial behavior (SE = 0.0049, 95% CI (0.0222, 0.0413)) were all significant mediators.

#### Childhood abuse to PLEs

The effect of childhood abuse on PLEs was 0.1598 (SE = 0.0067, 95% CI [0.1426,0.1729] and the effect of Childhood abuse on PLEs after control wisdom was 0.1306 (SE = 0.0069, 95% CI [0.1117, 0.1440]) on, which did not cover zero, wisdom partly mediated the relationship between childhood abuse and PLEs. The mediating model explained 18.27% of the model effect. Consistently with previous, decisiveness (SE = 0.0010, 95% CI (0.0048, 0.0087)), emotional regulation (SE = 0.0024, 95% CI (0.0239, 0.0332)) and prosocial behavior (SE = 0.0027, 95% CI (0.0114, 0.0219)) were all significant mediators.

## Discussion

To the best of our knowledge, this is the first study to determine the role of wisdom on the relationship between CT and PLEs in a multicenter non-clinical student sample. Specifically, we reported three important findings, first, participants with more trauma reported higher score of PLEs and lower score of wisdom. Second, wisdom was found significantly negatively correlated with CT and PLEs. Third, we found that the association between CT and the occurrence of PLEs can be mediated by decreased wisdom level.

The relationship between childhood maltreatment and psychotic symptoms has been well-established. In line with previous studies [25, 68–70], we replicated the positive correlation between CT and PLEs in Chinese university students, further validating the connection between early childhood trauma and psychosis risks. Moreover, we identified the mediating role of decisiveness, prosocial behavior, emotional regulation and total score of wisdom in the relationship between CT and PLEs. All in all, our results support the assumption that deficits in wisdom are significantly impact on the pathway between CT and PLEs, which are in line with some previous studies [34, 71]. The research expanded on previous findings by investigating the mediating role of wisdom in a large multicenter sample of Chinese university students. What’s more, in accord with previous research [72–75], we discovered that specific aspects of wisdom were significant mediators in the relationship between CT and PLEs. Since our study also revealed that wisdom was inversely correlated with PLEs, we considered that exposure to traumatic events in childhood leads to psychotic symptoms by reducing individual cognitive function or emotion regulation, further impact on wisdom level. Our results implicated that the experience of traumatic life events during childhood may have a strong influence on the wisdom level in adulthood, thus increasing the frequency of psychotic experiences.

The significant effect of the wisdom alleviated the impact of CT on PLEs can be understood by an ancient mechanism [30, 49]. The component of wisdom, such as prosocial behavior and emotional regulation, improves with critical life experiences, which contributes to increase wisdom, further protecting individual mental health and reducing the occurrence of mental disorders. Treatment goals of trauma therapies or intervention for people at high risk of mental disorders is the raising of wisdom.

Regarding the model of childhood neglect - wisdom - PLEs and childhood abuse - wisdom - PLEs, we both found significant partial mediation. Compared to childhood neglect, we observed that childhood abuse was particularly associated with PLEs, indicating that the type of trauma could be relevant for the development of PLEs.

Our findings may have important clinical implications. Considering that wisdom is the quality of a possible trait can be enhanced [27], which may be beneficial for enhancing the mental health of population with traumatic experiences in which all markers should be extensively assessed. Consequently, targeted interventions to enhance wisdom may have the potential to reduce the impact of early childhood adversity on the emergence of PLEs, and even lower the social burden of psychosis considering PLEs as a risk factor to clinical psychosis.

Limitations should be considered in our research. First, in this research, 38 participants were excluded due to missing data, which may cause potential selection bias. Second, the cross-sectional nature of our study limited the inference of causal relationship, further longitudinal design is warranted to verify the role of wisdom in psychosis risk studies. Furthermore, the self-report measures may limit the precise positioning of PLEs.

## Conclusions

To conclude, our findings indicate that wisdom could play a role in psychosis proneness among non-clinical college students exposed to childhood trauma, and further emphasize the relevance of assessing and boosting wisdom when working with individuals who are in a vulnerable stage for mental health problems.

## Data Availability

The datasets used and/or analyzed in the current study are available from corresponding authors on reasonable request.

## Declarations

### Note

CTQ-28-Sum (Cumulative Trauma) – total score of the CTQ; Neglect – a summary of physical and emotional neglect subscales of the CTQ; Abuse – a summary of sexual, physical and emotional abuse subscales of the CTQ; SD-WISE- total score of the SD-WISE; CAPE-P15- total score of the frequency subscales of the PLEs.

## Abbreviations

CT: Childhood trauma.
PLEs: Psychotic-like experiences.
SD-WISE: the San Diego Wisdom Scale.
CTQ-28: the Childhood Trauma Questionnaire.
CAPE-15: the 15-item Positive Subscale of the Community Assessment of Psychic Experiences.
SAWS: the Self-Assessed Wisdom Scale.
3D-WS: the Three-Dimensional Wisdom Scale.
PI: Persecutory Ideation.
BEs: Bizarre Experiences.
PAs: Perceptual Abnormalities.
CI: Confidence interval.
